# DNA methylation profiles correlated to striped bass sperm fertility

**DOI:** 10.1186/s12864-018-4548-6

**Published:** 2018-04-10

**Authors:** L. Curry Woods III, Yaokun Li, Yi Ding, Jianan Liu, Benjamin J. Reading, S. Adam Fuller, Jiuzhou Song

**Affiliations:** 1 Department of Animal and Avian Sciences, University of Maryland, College Park, MD 20742 USA; 2 College of Animal Science, South China Agricultural University, Guangzhou, GD 510642 China; 3 Department of Applied Ecology, North Carolina State University, Raleigh, NC 27695 USA; 4 HKD Stuttgart National Aquaculture Research Center, Agricultural Research Service, US Department of Agriculture, Stuttgart, AR 72160 USA

**Keywords:** Striped bass, Semen, Fertility, Methylation, Aquaculture

## Abstract

**Background:**

Striped bass (*Morone saxatilis*) spermatozoa are used to fertilize in vitro the eggs of white bass (*M. chrysops*) to produce the preferred hybrid for the striped bass aquaculture industry. Currently, only one source of domestic striped bass juveniles is available to growers that is not obtained from wild-caught parents and is thus devoid of any genetic improvement in phenotypic traits of importance to aquaculture. Sperm epigenetic modification has been predicted to be associated with fertility, which could switch genes on and off without changing the DNA sequence itself. DNA methylation is one of the most common epigenetic modification types and changes in sperm epigenetics can be correlated to sub-fertility or infertility in male striped bass. The objective of this study was to find the differentially methylated regions (DMRs) between high-fertility and sub-fertility male striped bass, which could potentially regulate the fertility performance.

**Results:**

In our present study, we performed DNA methylation analysis of high-fertility and sub-fertility striped bass spermatozoa through MBD-Seq methods. A total of 171 DMRs were discovered in striped bass sperm correlated to fertility. Based on the annotation of these DMRs, we conducted a functional classification analysis and two important groups of genes including the WDR3/UTP12 and GPCR families, were discovered to be related to fertility performance of striped bass. Proteins from the WDR3/UTP12 family are involved in forming the sperm flagella apparatus in vertebrates and GPCRs are involved in hormonal signaling and regulation of tissue development, proliferation and differentiation.

**Conclusions:**

Our results contribute insights into understanding the mechanism of fertility in striped bass, which will provide powerful tools to maximize reproductive efficiencies and to identify those males with superior gametes for this important aquaculture species.

**Electronic supplementary material:**

The online version of this article (10.1186/s12864-018-4548-6) contains supplementary material, which is available to authorized users.

## Background

The striped bass (*Morone saxatilis*) is a high value, high quality aquaculture species and contributes over $48 M annually to the US aquaculture industry. Striped bass sperm are routinely used to fertilize in vitro eggs of white bass (*M. chrysops*) to yield the hybrid striped bass which is the fourth largest finfish species by value in the US. Currently, fingerlings of the hybrid species are not available to producers on a year-round basis due to the lack of established, genetically improved commercial brood stocks with adequate methods for the distribution of their germplasm. Additionally, survival of the striped bass embryos and fry from fertilization to fingerlings for grow-out is often less than 25%, leading to negative effects on the profitability of the industry [1]. Significant efforts have been made during the past decade to alleviate the reproductive and genetic inefficiencies of the hybrid striped bass aquaculture industry, including the development of effective methods to cryopreserve striped bass sperm [1–3], selective breeding of both moronid progenitor species used to produce the industry standard hybrid [4] and the development of transcriptome and genome resources [5, 6]. The gamete and embryo quality of striped bass might also be improved via epigenetic modifications of spermatozoa genomic DNA.

Epigenetic modification can switch genes on and off without changing the DNA sequence itself [7]. DNA methylation, histone modification and RNA-associated repression are the three principle epigenetic mechanisms to trigger and maintain gene silencing, and dysfunction of these processes could cause abnormal expression of certain genes leading to pathology. It is broadly accepted that DNA methylation is a significant factor in the regulation of gene expression [8], which is furthermore involved in the regulation of most biological processes including: embryonic development, genetic imprinting, transcription, chromatin structure, and chromosome stability [9–13]. In mammals, cytosine methylation of the CpG dinucleotide is the main DNA methylation type, which could influence transcription factor binding, indirectly inhibiting the gene expression by recruitment of the methyl-CpG binding domain (MBD) protein that can change chromatin structure [14]. In pigs, DMRs in the promoter may be correlated with the repression of some obesity-related genes [15]. While genome-wide DNA methylation profiles of organisms including: avian, porcine, bovine and human have been reported [16–19]; little is known about DNA methylation profiles of fish in general and nothing has been reported regarding the DNA of striped bass spermatozoa and its correlation to fertility.

Male fertility relies on the characterization of sperm cells, including gene expression, DNA methylation, and histone modification. Fertility is crucial in the regulation of reproduction, which is believed to be influenced by epigenetic phenomena including DNA methylation. However, molecular details on mechanisms by which sperm fertility is regulated by DNA methylation in vertebrates are poorly defined. Therefore, in our present study, we utilized the sperm from striped bass males with known differences (via egg controls) in their rate of fertility to characterize the methylation profiles of these striped bass sperm by direct deep sequencing, which allowed us to correlate these differing DNA methylation profiles with sperm fertility. These results constitute a valuable resource for future functional studies and the discovery of potential epigenetic biomarkers for selecting male striped bass with excellent fertilization performance. The findings of this study will be significant for identifying potential mechanisms leading to improved fertility rates of striped bass; which could provide a means to screen male broodstock for individuals that produce high fertility gametes and/or to modify epigenetic imprinting for production of optimal gametes.

## Methods

### Experimental fish and sperm sample collection and preparation for DNA extraction

The fish used for this study were part of the *National Program for Genetic Improvement and Selective Breeding for the Hybrid Striped Bass Industry* research program at the Harry K. Dupree Stuttgart National Aquaculture Research Center (HKD-SNARC), Agricultural Research Service (ARS), US Department of Agriculture, Stuttgart, Arkansas and North Carolina State University (NCSU) Pamlico Aquaculture Field Laboratory, Aurora, North Carolina (NCSU-PAFL). Animal care and experimental protocols were approved by the HKD-SNARC Institutional Animal Care and Use Committee (IACUC) and conformed to ARS Policies and Procedures 130.4 and 635.1. Broodstock for this study consisted of domesticated four year-old female white bass (WB) and domesticated five year-old male striped bass (SB). Domesticated broodstock were obtained as fingerlings from the NCSU-PAFL and reared to maturity at the HKD-SNARC. The domesticated WB line originally established at the NCSU-PAFL by outcrossing WB from Lake Erie with fish from the Tennessee River, has been domesticated for over 8 generations. The domesticated SB line was originally established at NCSU-PAFL by outcrossing SB from six stocks (Canadian, Hudson River, Roanoke River, Chesapeake Bay, Santee-Cooper Reservoir, and Florida-Gulf of Mexico) and has been domesticated for over 6 generations.

Broodstock were conditioned in outdoor 1-acre ponds and brought into cold bank facilities one week prior to spawning. Female WB were given 75 μg GnRH*a* Ovaplants®(Syndel Laboratories, Cat. No. 13460), injected into the dorsal musculature. Fish to be spawned were chosen in an arbitrary manner based on those that were conditioned and available at the time of spawning. All fish were strip spawned, with eggs from each WB female separated into two labeled spawning bowls where sperm from two SB males would be used to complete fertilization to produce conspecific hybrid striped bass (one male SB for each WB bowl with eggs from the same female serving as the control for female fertility). Well-water was added to the egg/sperm mixture to activate sperm. The eggs and sperm were allowed to stand in the well water for approximately 2 min and then gently poured into McDonald hatching jars until fertile eggs began to hatch (approximately 36-40 h post-fertilization). The fertilization rate was determined by examining 200-300 eggs, approximately two hours post-fertilization, for signs of development using a dissecting light microscope (10X). Eggs that showed no development were assumed to be unfertilized or dead (arrested in embryonic development).

We selected four representative high-fertility (HF) male striped bass (animal ID: 1922, 1927, 1929 and 1930, with fertility rates of 94%, 87%, 76%, and 76% respectively) and three representative sub-fertility (SF) male striped bass (animal ID: 1916, 1917 and 1938, with fertility rates of 11%, 0%, and 0% respectively) from the population of fish spawned. The white bass egg duplication allowed us to de-select any males that were part of a given spawning where no eggs were fertilized from either WB bowl (i.e., presumption of poor egg quality or female infertility). All fish were anaesthetized to initial loss of equilibrium using 30 ppm Tricaine-S (Western Chemical, Ferndale, WA, USA). Gametes were stripped using light abdominal pressure, fish were then returned to well-oxygenated water containing a 1% NaCl solution to minimize stress in a recovery tank before being returned to their culture tanks. Aliquots of striped bass semen were collected at the time of spawning for DNA extraction and sequencing and snap frozen in liquid nitrogen. Briefly, approximately 1 mL of semen was put into a sterile conical tube and was then subsequently diluted with isosmotic (350 mOsmol/Kg) Striped Bass Extender [20] to provide an extended striped bass semen mixture containing approximately one billion sperm cells per 100 μL of extended semen. One mL of this mixture was pipetted into a sterile, RNAse free tube and immediately plunged into liquid nitrogen. It was kept frozen until a later time when it was thawed to extract and purify the DNA from the striped bass sperm. Care was taken to avoid any contact of water with semen prior to collection and freezing.

### DNA extraction and MBD-Seq library construction

MBD-seq method was employed to identify methylated DNA regions in striped bass sperm. Briefly, we extracted the genomic DNA from sperm using the OmniPrep™ for Tissue kit (G Biosciences, Cat. NO: 786-395) and purified the DNA samples using the MinElute PCR Purification Kit (Qiagen, Cat. NO: 28006).The purified DNA concentration was measured by the Qubit dsDNA Broad-Range Assay (Invitrogen, Cat. NO: Q32850) and each sample was adjusted to 0.1 μg/μl with a final volume of 55 μl and then sheared into 300–500 bp fragments. The Methyl Cap Kit (Diagenode, Cat. NO: C02020010) was used to obtain the methylated DNA, according to the manufacture’s instructions.

The MBD-Seq library was constructed as follows. The NEBNext End Repair Module (NEB, Cat. NO: E6050S) was used for end repair of the fragmented, methylated DNA. After the 3′ poly “A” was added, a pair of Solexa adaptors (Illumina) was ligated to the repaired ends using T4 ligase (Promega, Cat. NO: M1801). The ligated products were then electrophoresed through 2% agarose gels and the fragments, ranging from 200 to 500 bp, were purified usinga Quick Gel Extraction Kit (QIAGEN, Cat. NO: 28704). We then enriched the purified DNA templates through PCR (the PCR program was as follows: 98 °C for 30s; 98 °C for 10s, followed by 60 °C for 30s and 72 °C for 10s, with 22 cycles; followed by the final 72 °C for 5 min), purified the PCR products using a MinElute PCR Purification Kit (QIAGEN, Cat. NO:28004) and then measured the concentration of the library using the Qubit Assay (Life Technology, Cat. NO: Q32850). The MBD-Seq library was sequenced on theSolexa 1G Genome Analyzer (Illumina) platform following the specifications provided by the manufacturer.

### MBD-Seq data analysis

The quality of the raw short reads was evaluated employing FastQC,a web-based software that provides a thorough examination of the reads. Then, Bowtie was used to align the reads to the reference genome established and maintained by the Reading laboratory: http://appliedecology.cals.ncsu.edu/striped-bass-genome-project/data-downloads/. The draft striped bass genome sequence assembly is 585 million bases (585 Mb) and comprised of 35,010 contigs, with a GC content of 40.0%, similar to that of the congeneric white bass (39.5%) (Reading et al., *unpublished data*). The assembly has a CEGMA completeness score of 86.29% (partial) and 70.56% (complete), indicating that the complete genome is probably 600-700 Mb in size. The MAKER annotation pipeline was used to identify 27,485 protein coding genes.

During the quality filtering step, we trimmed the first 15 bases of each short read to maintain high sequence quality score, which resulted in 35 bp tags. For our analysis, a combination of procedures available in SAMtools and BEDtools were applied for the data filtration and format conversion.

The Model Based Analysis of ChIP-Seq (MACS) was implemented for peaks identification of each sample. This software utilized a dynamic Poisson distribution to effectively catch the local bias, improving the reliability of the prediction. After creating a contrast between conditions, the R package DiffBind runs an edgeR analysis with a false discovery rate (FDR) < 0.1 to call the differentially methylated regions (DMRs). Then, the ChIPpeakAnno package was used for the genomic annotation of the previously identified DMRs. This software provides information about the distance, relative position, and overlaps for the inquired feature.

### Pyrosequencing for MBD-Seq validation

Equal amounts of DNA from the samples of each group were pooled together, serving as the template for the bisulfite conversion and the bisulfite PCR. Sodium bisulfite conversion reagents were used to treat 500 ng of each DNA pool (Methyl EdgeTM Bisulfite Conversion System, Promega). The DMRs for validation were randomly selected from the bioinformatics analysis results. The PCR primers were designed with PSQ Assay Design software (Biotage, Swedan) and shown in Additional file 1: Table S1.

Each pyrosequencing reaction contained 10-20 ng bisulfite converted DNA, and pyrosequencing methylation analysis was performed utilizing the Pyro Q-CpG system (PyroMark ID, Biotage). Briefly, the PCR products were bound to Streptavidin coated Sepharose beads (GE Healthcare Bio-sciences AB, Sweden). Then, the beads were purified in 70% ethanol for 5 s, denatured in Denature buffer (Biotage) for 5 s, and washed with washing buffer (Biotage) for 10 s in the pyrosequencing Vacuum Prep Tool (Biotage). Next, 0.5 mM sequence primer was annealed to the purified single-stranded PCR product and pyrosequencing was carried out using the Pyro Q-CpG system. The methylation level was expressed for each cytosine locus on CpG sites as the percentage of ^m^C/(^m^C + C), where“^m^C” is methylated cytosine and “C” is unmethylated cytosine. Non-CpG cytosine residues were used as internal controls to verify bisulfite conversion. Finally, DNA methylation level was obtained.

### DMR gene annotation enrichment analysis

The positional information of DMR regions was used to identify neighboring predicted genes on each of the genome assembly contigs. A list of the predicted genes was then compiled and identities of the informative loci were used to retrieve official gene symbols that were submitted to DAVID Gene Functional Classification for Gene Ontology (GO) Class enrichment analysis [21]. Default parameters were used for DAVID with the complete gene list serving as the background. Functional characteristics of the annotated genes associated with DMRs also were evaluated, focusing mainly on GO biological process, cellular component, and molecular function that were enriched overall in the gene set.

## Results

### Methylation analysis

**Fig. 1:**
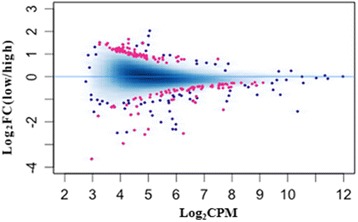
Differentially methylated regions (DMRs) between high-and sub-fertility striped bass sperm samples.The methylation analysis plot shows in red the DMRs obtained with a false discovery rate of < 0.1. The blue dots represent the methylated regions without difference between high-and sub-fertility groups. “FC” indicates fold-change and “CPM” indicates counts per million

**Fig. 2:**
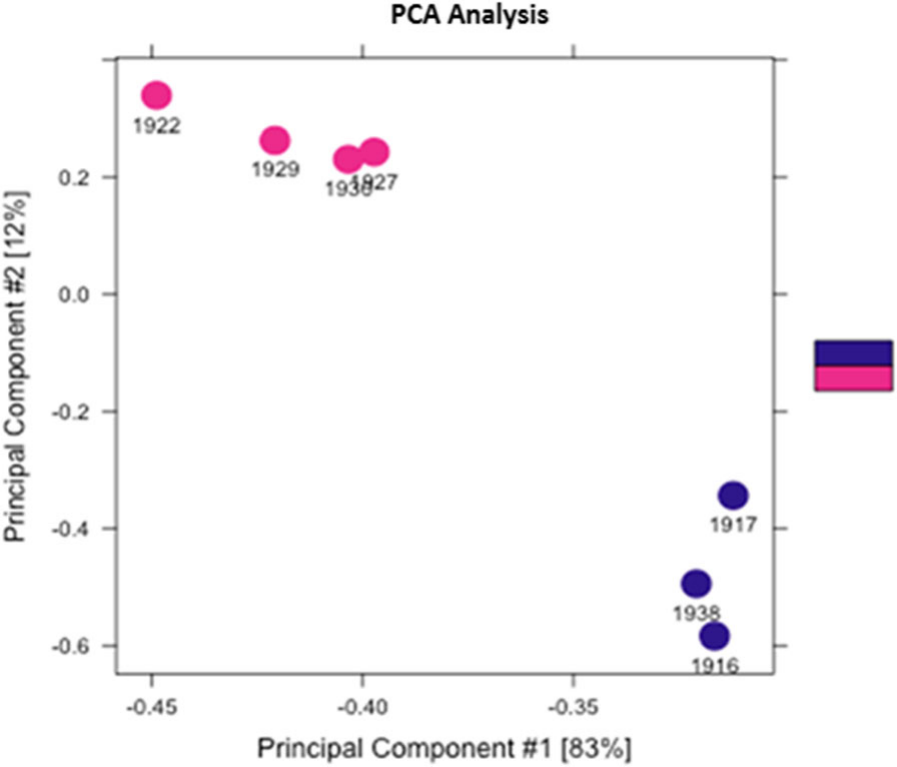
Principle component analysis (PCA) of striped bass sperm from high- and sub-fertility groups based on identified differentially methylated regions (DMRs). The red color represents the high-fertility striped bass; the blue color represents the sub-fertility striped bass

Of the seven experimental samples, the sequence alignment scores were 84.83%, 85.32%, 85.10%, 84.72%, 85.82%, 85.97%, and 85.51% (Additional file 2: Figure S1). All of these scores were greater than 80%, with no exceptional differences between any of the individuals or experimental fertility groups. For the statistical analysis, we applied the DESeq2 package implemented in R to detect the DMRs in the sperm samples of high-fertility and sub-fertility groups. The threshold False Discovery Rate (FDR) < 0.1 was used as the nominal level of significant difference. Following this strategy, we found 171 DMRs between the two groups (Additional file 3: Table S2), which are presented in Fig. 1. Based on the identified DMRs, a cluster analysis using principal components of our experimental samples was performed and illustrated in Fig. 2. These results demonstrate the consistency of condition within the samples originating from either the high- or sub-fertility male striped bass.

### DMR gene annotation enrichment analysis

A characterization of annotated genes was subsequently performed to identify the functions of genes, potentially regulated by the DMRs, and their roles in fertility of striped bass (Additional file 4: Table S3). Protein coding genes present on the 171 contigs that contained DNA methyl markers were annotated from the 27,485 predicted gene loci in the striped bass genome. Fifty-five (55) contigs contained no predicted gene loci (32.2% of 171). The remaining 116 contigs contained one or more predicted gene loci with 68 annotated (58.6%) and 48 unknown or novel (41.4%). The contig N50 of the genome sequence assembly is 29,981 bp, indicating that on average the gene loci are within this distance or less from the putative regulatory methyl markers. The number of markers located upstream or downstream of predicted genes was roughly 33% each and the remaining third of DMRs were located within predicted protein-coding gene regions. In some cases, DMRs located within a gene region spanned into either the upstream or downstream non-coding regions. Overall the average length of each DMR was 476 ± 378 bp (standard deviation). A genome-wide analysis of predicted CpG islands was not performed on the striped bass genome sequence as the assembly is still considered a draft.

The official gene symbols of 68 informative loci were submitted for GO Class enrichment analysis and generally, the genes were classified into two categories: the WDR3/UTP12 family and the G protein-coupled receptors (GPCR) family. The enriched groups of genes are shown in Table 1. The functional characteristics of the genes based on biological process, cellular component, and molecular function that were enriched in the gene set also are provided in Table 2. The most significant GO terms were eye development, gastrulation, cell division and phosphoprotein-phosphatase activity. Most of the above processes discovered in our study were related to organ development, DNA replication and enzyme activity.

**Table 1:** David Gene Ontology (GO) enrichment analysis of those genes associated with differentially methylated regions in striped bass sperm of high- and sub-fertility

Symbol	Gene	Fold- Methylation
Group 1: WD repeat containing proteins. Enrichment Score: 1.00		
*wdr3*	WD repeat domain 3	+ 1.188
*lrba*	LPS-responsive vesicle trafficking, beach and anchor containing	+ 1.100
*wdr65*	WD repeat domain 65	+ 1.278
*wsb2*	WD repeat and SOCS box-containing 2	−1.342
*wipi2*	WD repeat domain, phosphoinositide interacting 2	−0.479
Group 2: G-protein-coupled receptors. Enrichment Score 0.38		
*ffar2*	free fatty acid receptor 2	−0.427
*gpr128*	G protein-coupled receptor 128	−0.503
*nmbr*	neuromedin B receptor	+ 1.091
*p2ry14*	purinergic receptor P2Y, G-protein coupled, 14	−0.342

A positive value under fold-methylation indicates a higher methylation rate in sub-fertile individuals and a negative value indicates a higher methylation rate in high-fertility individuals

**Table 2:** Gene Ontology (GO) terms enriched with the genes corresponding to the differentially methylated regions (*P* < 0.05) in striped bass sperm of high- and sub-fertility

Biological Process
GO:0001654 eye development *P* = 1.91 × 10^− 4^
*bbs9* (− 0.919 fold methylation, DMR downstream of gene), *vangl2* (+ 1.130 fold methylation, DMR within gene)*, nfkb1* (+ 1.199 fold methylation, DMR within gene)*, lamc1* (− 0.796 fold methylation, DMR within gene)*, axin1* (+ 0.772 fold methylation, DMR within gene)
GO:0007369 gastrulation *P* = 1.56 × 10^− 2^
*bbs9* (− 0.919 fold methylation, DMR downstream of gene)*, vangl2* (+ 1.130 fold methylation, DMR within gene)*, bcl2l10* (+ 0.987 fold, DMR downstream of gene)
GO:0051301 cell division *P* = 1.63 × 10^− 2^
*cdc6* (− 0.576 fold, DMR downstream of gene)*, babam1* (+ 1.174 fold, DMR downstream of gene)^a^ *nuf2* (+ 1.278 fold, DMR downstream of gene)*, cntrl*(− 0.481 fold, DMR downstream of gene)
GO:0007257 activation of JUN kinase activity *P* = 2.49 × 10^− 2^
*mapk8ip3* (− 2.553X fold, DMR downstream of gene)^a^*, axin1* (+ 0.772 fold, DMR within gene)
GO:0006261 DNA-dependent DNA replication *P* = 4.44 × 10^− 2^
*rfc5* (− 1.342 fold, DMR downstream of gene)*, rfc4* (− 0.565 fold, DMR downstream of gene)
Cellular Component
GO:0005663 DNA replication factor C complex *P* = 2.54 × 10^− 2^
*rfc5* (− 1.342 fold, DMR downstream of gene), *rfc4* (− 0.565 fold, DMR downstream of gene)
GO:0005739 Mitochondrion *P* = 4.82 × 10^− 2^
*mrps34* (− 2.553 X fold, DMR downstream of gene)^a^*, spg7* (+ 1.038 fold, DMR downstream of gene)*, gtpbp3* (+ 1.174 fold, DMR downstream of gene)^a^, *pgam5* (+ 1.065 fold, DMR upstream of gene), *echdc3* (− 0.366 fold, DMR downstream of gene)*, apex1* (− 0.315 fold, DMR downstream of gene)*, bcl2l10* (+ 0.987 fold, DMR downstream of gene)
Molecular Function
GO:0004721 phosphoprotein phosphatase activity *P* = 1.81 × 10^− 2^
*ppm1e* (− 0.413 fold, DMR within gene), *ppp3cca* (− 1.002 fold, DMR downstream of gene)^a^, *pgam5* (+ 1.065 fold, DMR upstream of gene), *ptpro* (− 0.288 fold, DMR within gene)
GO:0004222 metalloendopeptidase activity *P* = 1.90 × 10^− 2^
*adam28* (− 1.002 fold, DMR within gene)^a^, *adamts6* (+ 1.032 fold, DMR upstream of gene), *spg7* (+ 1.038 fold, DMR downstream of gene), *afg3l2* (− 0.321 fold, DMR within gene)
GO:0008237 metallopeptidase activity *P* = 2.68 × 10^− 2^
*adam28* (− 1.002 fold, DMR within gene)^a^, *adamts6* (+ 1.032 fold, DMR upstream of gene), *spg7* (+ 1.038 fold, DMR downstream of gene), *afg3l2* (− 0.321 fold, DMR within gene)
GO:0030159 receptor signaling complex scaffold activity *P* = 3.45 × 10^− 2^
*mapk8ip3* (− 2.553X fold, DMR downstream of gene)^a^, *axin1* (+ 0.772 fold, DMR within gene)
GO:0003689 DNA clamp loader activity *P* = 3.45 × 10^− 2^
*rfc5* (− 1.342 fold, DMR downstream of gene), *rfc4* (− 0.565 fold, DMR downstream of gene)

The approved gene names are provided for each GO class along with methylation status in the fertility groups and DMR position in relation to the protein coding portion of each gene. A positive value with fold-methylation indicates a higher methylation rate in sub-fertile individuals and a negative value indicates a higher methylation rate in high-fertility individuals. ^a^The following genes are located nearby on the same contigs: *mapk8ip3* and *mrps34*; *gtpbp3* and *babam1*; *adam28* and *ppp3cca*

### Validation of the DMRs by pyrosequencing

**Fig. 3:**
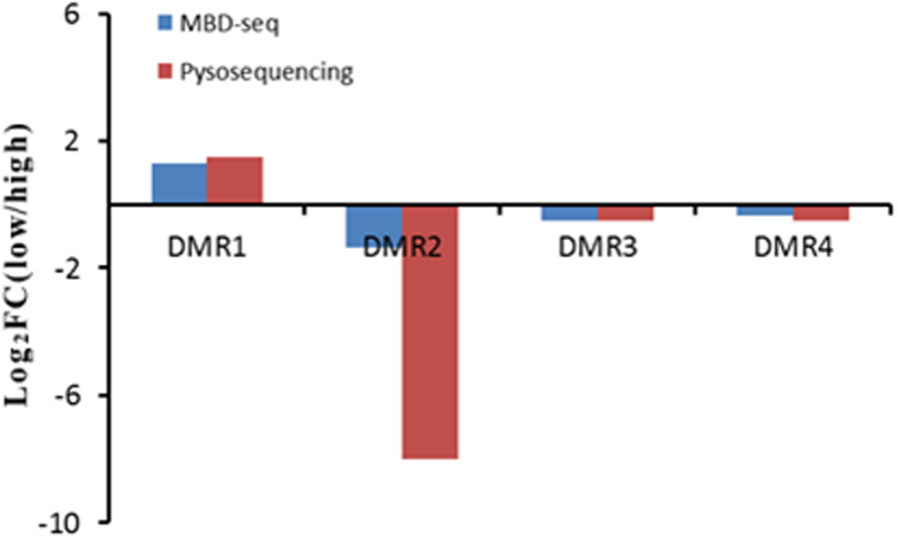
Validation of MBD-Seq results through pyrosequencing. “FC” indicates fold-change; “low” indicates the methylation level in sub-fertility group; “high” indicates the methylation level in high-fertility group

In order to assess the reliability and accuracy of DMRs detected by MBD-Seq, 4 DMRs were randomly selected for orthogonal validation. The calculated methylation levels are shown in Fig. 3. We found that the methylation level trend of all the 4 regions were generally in agreement with MBD-Seq analysis. The pyrosequencing employed only a segment of the methylation region to perform the validation, thus the magnitude of methylation differences in the validation results are not exactly the same as the MBD-Seq analysis and this is to be expected.

## Discussion

DNA methylation is one of the most common epigenetic modifications, which is distinguished by a stable methyl group addition to cytosine, most often in DNA regions enriched with CG [22]. It was suggested that the DNA methylation level of human sperm was associated with the concentration and motility of the spermatozoa, suggesting a relationship between the DNA methylation modification and the fertility rate of the sperm [20]. In buffalo bulls, between high fertile and sub-fertile individuals, the methylation level of 96 genes related to spermatogenesis, embryonic development, and capacitation was significantly different [21]. The performance of horse semen, shown to decrease after cryopreservation, has been correlated with a concomitant change in DNA methylation of the spermatozoa demonstrating the possible effects of sperm DNA methylation on the quality of semen [23]. Collectively, these studies illustrated that aberrant sperm DNA methylation could result in reduced fertility or reproductive efficacy and suggested that DNA methylation might be a valuable consideration for the assessment of semen quality. We developed the hypothesis that DNA methylation is a major regulator of sperm function, influencing the fertility rate of the sperm in striped bass. Gene expression was not evaluated in the present study, thus it is unclear if these methylation patterns influence gene expression in the male striped bass during spermatogenesis or if they are somehow involved in the pronucleus formation or other processes following fertilization such as genome compatibility between striped bass and white bass. Given the timing of the mid-blastula transition in striped bass [24], it is unlikely that the sperm methylation patterns influence embryonic gene expression leading to embryo mortality and hence low sperm fertility as this occurs several hours after fertilization and fertility evaluations in the present study were conducted approximately two hours post-fertilization. Therefore, embryos produced using sub-fertile sperm had already arrested development prior to the onset of embryonic gene expression. Nonetheless, the DMRs are predictive of fertility rate in male striped bass (Fig. 2) and thus could serve as important predictors of semen quality.

**Fig. 4:**
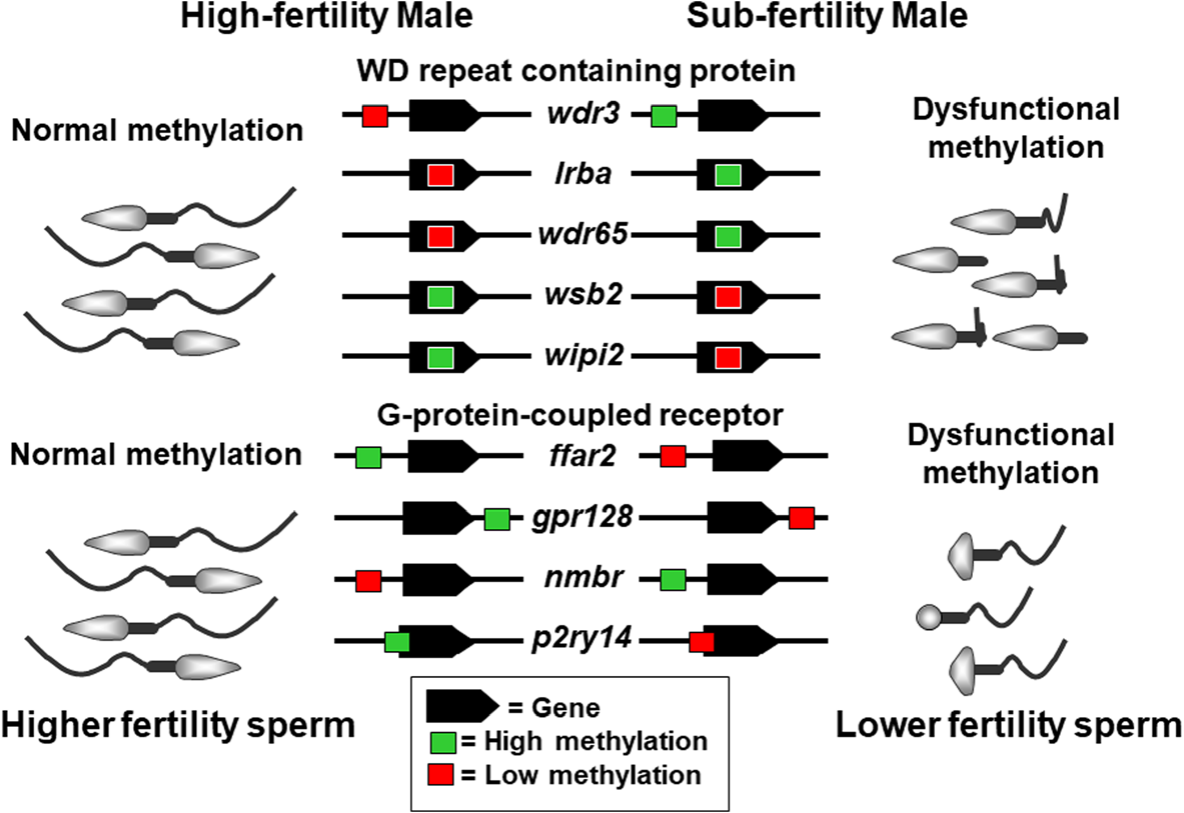
Hypothetical models of sperm fertility in striped bass influenced by differentially methylated regions associated with critical reproductive genes. Genes representing the WD repeat containing protein and G-protein-coupled receptor families are represented as indicated in Table 1. The relative positions (although not drawn to scale) of methylated regions along with methylation status of males from the high- and sub-fertility groups are shown

In the present study, we discovered 171 DMRs between highly-fertile and sub-fertile striped bass sperm samples. Of these DMRs, 4 were randomly selected for orthogonal validation by pyrosequencing, which was in agreement with the MBD-Seq results and supporting the identification of these 171 DMRs as robust. Among the annotated genes associated with the DMRs, we detected enrichment of two major gene families considering their functions: WDR3/UTP12 family and GPCR family (Table 1). The WDR3/UTP12 family has a strong enrichment score (1.00). This is a large family of proteins characterized by ‘WD repeats’ also called transducin repeats, GH-WD repeats, or WD 40 repeats. They are minimally conserved domains of approximately 40-60 amino acids that begin with a glycine-histidine dipeptide 11-24 residues from the N-terminus and terminate with a tryptophan-aspartic acid dipeptide (WD) at the C-terminus [25]. The WD repeat is considered to be responsible for protein-protein interactions and WD repeat containing proteins are represented in the sperm flagellum of vertebrates [26]. Several cases of male infertility have been associated with dysfunctional WD repeat containing proteins including asthenozoospermia, characterized by reduced forward sperm motility in humans and mice [27, 28]. There are 5 methylated genes in the sperm of striped bass that encode WD repeat containing proteins and that also are associated with fertility (*wdr3*, *lrba*, *wdr65*, *wsb2*, and *wipi2*). Three of these genes have enhanced methylation status in sub-fertile males, whereas 2 of them are methylated in highly fertile individuals (Table 1). With the exception of *wdr3*, in which the DMR is located upstream of the coding region, the DMRs for the remaining 4 genes are included within the predicted gene site. Therefore, the methylation patterns on this particular group of genes may negatively influence expression leading to the formation of sperm flagella with reduced swimming capability resulting in the lower fertilization rates by some male striped bass (Fig. 4).

G-protein-coupled receptors (GPCRs) are one of the largest and most diverse protein families whose primary function is to transduce extracellular stimuli into intracellular signals through interaction of their intracellular domains with heterotrimeric G proteins [29]. In our study, we detected 4 methylated genes in the sperm of striped bass that encode GPCRs and that also are associated with fertility, which were *ffar2*, *gpr128*, *nmbr*, and *p2ry14*. Three of these genes are methylated in the males from the high-fertility group and one is methylated in the sub-fertility group (Table 1). The *ffar2* gene is associated with short-chain fatty acid receptors that modulate protein phosphorylation status, insulin secretion, and inflammatory response. Also, during pregnancy, *ffar2* plays an important role in metabolic homeostasis [30, 31]. In addition, PI3K/Akt (phosphatidylinositol 3-kinase) and EPK2/PRKD3 (serine- and threonine-specific protein kinase) signal transduction involved in diverse cellular pathways could also be influenced by *ffar2* [32]. In the striped bass, the *ffar2* gene has a DMR region located upstream of the protein coding site. As for *p2ry14*, it is the receptor of UD-glucose, with involvement in embryo implantation, suggesting a possible function in fertility [33, 34]. The striped bass *p2ry14* gene has a DMR located upstream and that spans into the protein coding gene region. Of several studies published about the function of *gpr128* and *nmbr,* nothing is related to fertility. This group of genes has a weaker enrichment score (0.38), but it is still significant. The striped bass *gpr128* has a DMR located downstream of the protein coding gene and *nmbr* has one located upstream. Virtually nothing is published about the direct relationship between these GPCR genes and infertility; however one function of this group may be regulation of tissue development, proliferation, and differentiation as well as response to hormonal cues from the reproductive endocrine axis. These may very well be novel indicators of animal infertility.

The remaining genes that were not considered enriched by DAVID may be involved in one or both of the above groups or show a novel function. The results of an overall GO functional characteristics analysis are provided in Table 2 and many of these GO processes related to genes with DMRs are unsurprising considering their general importance to reproduction and embryogenesis (e.g., eye development, gastrulation, cell division, and DNA replication). Also, the majority of these genes either contain the methylated region within the gene site or it is present downstream of the gene site, with few having methylation regions upstream of the transcription initiation site. Three pairs of genes assigned GO classes as listed in Table 2 are physically linked in the genome and the loci are co-localized within about 30 Kb on the same contig assemblies: *mapk8ip3* and *mrps34*; *gtpbp3* and *babam1*; *adam28* and *ppp3cca*. This observation is likely not coincidental and, based on Occam’s Razor, may represent co-regulation by methylation status with synergistic gene functions that may occur during spermatogenesis (or pathology thereof). These observations further support the integrity of the methylation analysis of sperm fertility. However, there was a difficulty in isolating RNA with high quality from sperm samples, gene expression validation of genes associated with DMRs will definitely be needed, which will further validate the hypothesis.

There is no obvious pattern of methylation in one family of genes that might indicate fertility status (Table 1 and Table 2). For example, not all of the genes representing a single gene family or GO process are methylated in high-fertility males. A similar finding has been made in striped bass females, whereby clear differences in the expression of particular ovary genes representing functional GO terms were not evident either, however it was evident that certain genes were differentially expressed in high and low fertility females [24, 35]. Our contention is that regulation of infertility as a pathological condition is very complex and that gene expression or methylation status should be considered holistically and genome-wide, not simply based on a restricted set of individual genes that may be up or down regulated (i.e., there is more than one way to make a “bad” egg). Figure 4 provides a simplistic example whereby male infertility may be caused by problems with sperm motility related to expression of the WDR3/UTP12 or GPCR families of genes identified here. We prefer to use the term “dysregulated” or “dysfunctional” (as opposed to up or down regulated) and the condition of infertility likely involves many different genes, not just the few highlighted here. Of these DMRs, many could be potential markers of sperm quality, especially when used collectively to screen sperm using machine learning methods that we similarly described for female striped bass reproductive performance [24] or those used for evaluation of other physiological responses in fishes [36–38].

It is important also to note that these functional gene categories are not mutually exclusive, nor do they represent all of the potential dysfunctions in the sperm (for example see Table 2 for multiple assignments of the same gene to different GO classes). This is not unexpected as many genes perform multiple cellular functions and some have limited evidence of function at present, however more data in the future will likely provide additional details of differential actions. For example, one striped bass male might be infertile or sub-fertile because of genes in the WDR3/UTP12 family, another male might be so because of genes in GPCR family, and a third male might be so because of genes in both groups or possibly due to genes not listed as enriched (or not evaluated for function because they have no present annotation information). We similarly demonstrate a complex interplay between genes representing many different pathways that are responsible for influencing fertility in female striped bass and the conclusions are that individual females may produce poor quality gametes for different reasons [24]. Importantly, the WDR3/UTP12 and GPCR families of genes are overrepresented among those genes with DMRs and likely are important in the sub-fertility of striped bass sperm observed in this study. Further investigation using gene expression analysis will be required to fully resolve this, alternatively, enrichment may be improved in a future computational analysis when more information becomes available in the GO databases.

Our results provide general information regarding the correlation of DNA methylation and fertility of male striped bass. We have identified several important genes possibly regulating the fertility performance of striped bass spermatozoa. However, we recognize that the DNA methylation analysis and the target gene functional classifications rely on a computational strategy. Thus, significant experimental validation in an independent sample set is still needed to verify the functions of these DMRs and particular genes in striped bass sperm. Analysis of the correlation between the transcriptome profiling and the DNA methylation is also required in order to prove transcriptional regulation by the DMRs and these studies are currently underway.

## Conclusion

In this study, we identified genes that may influence the fertility of male striped bass through genome-wide MBD-Seq. In total, 171 DMRs were discovered between high-fertile and sub-fertile striped bass spermatozoa. Based on the annotation of those DMRs, we performed a functional classification analysis and two important groups of genes were found that may possibly be related to the fertility performance of striped bass sperm: the WDR3/UTP12 and GPCR families. In conclusion, our results contribute insights into understanding the mechanism of how to improve or predict the fertility in striped bass and other vertebrate species.

## Additional files

Additional file 1: **Table S1.** Primers used for pyrosequencing validation of differentially methylated regions (DMRs) identified by MBD-Seq(XLS). (XLS 29 kb)

Additional file 2: **Figure S1.** Alignment scores (percent of total) of MBD-Seq short reads aligned to the striped bass genome. (PNG 343 kb)

Additional file 3: **Table S2.** Differentially methylated regions (DMRs) between high- and sub-fertility striped bass sperm at a strict false discovery rate (FDR) < 0.1 (XLS). (XLS 53 kb)

Additional file 4: **Table S3.** Annotation of the identified differentially methylated regions (DMRs) in striped bass sperm from high- and sub-fertility groups. Genes present on contigs with DMRs are indicated in the column to the left (DMRs) and are numbered according to the striped bass genome assembly files provided at http://appliedecology.cals.ncsu.edu/striped-bass-genome-project/data-downloads/. Additional information including start and end of the gene transcripts on each of the contigs, length of the DMR site (Width), methylation levels in fish from the sub-fertile (Conc_Low) and high-fertile (Conc_High) groups, methylation fold-difference between fertility groups (negative value indicates greater in the high fertility group in reference to the low fertility group), *p*-value, false discovery rate (FDR), gene name (Name), approved gene symbol (Gene), gene position on each of the contigs (Gene Position), and method of annotation (ID) either from the striped bass genome or BLAST of the National Center for Biotechnology Information (XLS). (XLS 91 kb)

## Abbreviations

DMRs: Differentially methylated regions
FDR: False discovery rate
GO: gene ontology
HF: High-fertility
MACS: Model Based Analysis of ChIP-Seq
MBD: Methyl-CpG binding domain
SB: Striped bass
SF: Sub-fertility
WB: White bass
