# Supplementary figures and images for: DNA methylation profiles correlated to striped bass sperm fertility
Source: BMC Genomics. 2018 Apr 10;19:244. doi: 10.1186/s12864-018-4548-6 (PMC5894188; doi:10.1186/s12864-018-4548-6)

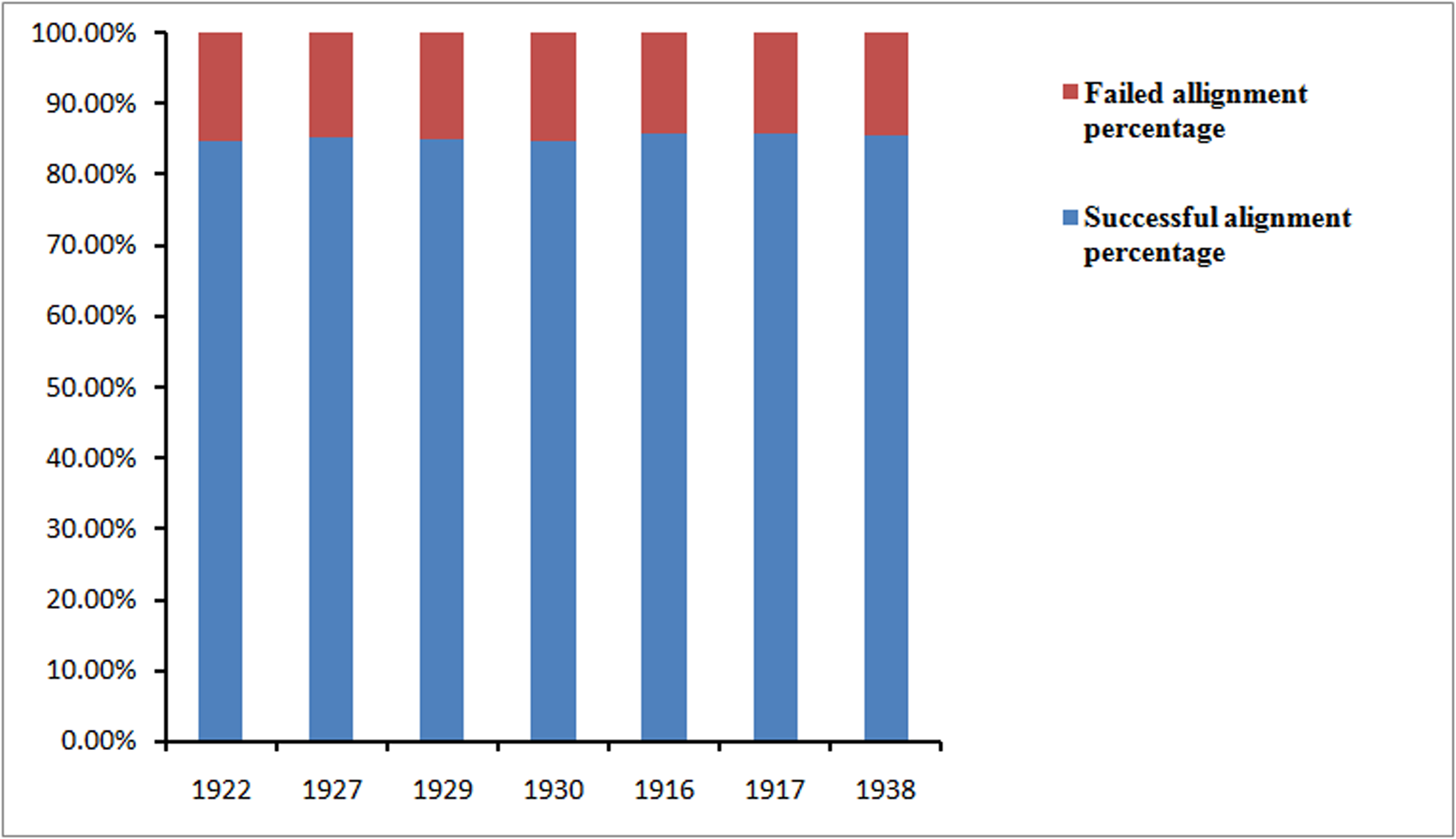

Supplement: Supplementary file 2 — Figure S1. Alignment scores (percent of total) of MBD-Seq short reads aligned to the striped bass genome. (PNG 343 kb) [file 12864_2018_4548_MOESM2_ESM.png]
